# Improving Newborn Respiratory Outcomes With a Sustained Inflation: A Systematic Narrative Review of Factors Regulating Outcome in Animal and Clinical Studies

**DOI:** 10.3389/fped.2020.516698

**Published:** 2020-10-29

**Authors:** Calista J. Lambert, Stuart B. Hooper, Arjan B. te Pas, Erin V. McGillick

**Affiliations:** ^1^The Ritchie Centre Hudson Institute of Medical Research, Melbourne, VIC, Australia; ^2^The Department of Obstetrics and Gynaecology, Monash University, Melbourne, VIC, Australia; ^3^Division of Neonatology, Department of Pediatrics, Leiden University Medical Center, Leiden, Netherlands

**Keywords:** sustained inflation, newborn respiratory outcome, clinical trials, animal models, ventilation, cardiorespiratory function, resuscitation and respiratory support

## Abstract

Respiratory support is critically important for survival of newborns who fail to breathe spontaneously at birth. Although there is no internationally accepted definition of a sustained inflation (SI), it has commonly been defined as a positive pressure inflation designed to establish functional residual capacity and applied over a longer time period than normally used in standard respiratory support (SRS). Outcomes vary distinctly between studies and to date there has been no comprehensive investigation of differences in SI approach and study outcome in both pre-clinical and clinical studies. A systematic literature search was performed and, after screening, identified 17 animal studies and 17 clinical studies evaluating use of a SI in newborns compared to SRS during neonatal resuscitation. Study demographics including gestational age, SI parameters (length, repetitions, pressure, method of delivery) and study outcomes were compared. Animal studies provide mechanistic understanding of a SI on the physiology underpinning the cardiorespiratory transition at birth. In clinical studies, there is considerable difference in study quality, delivery of SIs (number, pressure, length) and timing of primary outcome evaluation which limits direct comparison between studies. The largest difference is method of delivery, where the role of a SI has been observed in intubated animals, as the inflation pressure is directly applied to the lung, bypassing the obstructed upper airway in an apnoeic state. This highlights a potential limitation in clinical use of a SI applied non-invasively. Further research is required to identify if a SI may have greater benefits in subpopulations of newborns.

## Introduction

The importance of providing respiratory support during neonatal resuscitation cannot be overstated. Technological advances within this field have significantly improved survival for newborns who either do not spontaneously breathe at birth or need support to maintain spontaneous breathing, most commonly due to prematurity. Historically, these advances came at the cost of ventilator-induced lung injury, a collection of related lung injuries that lead to chronic lung disease (1, 2). More recently, clinical practice has moved toward non-invasive respiratory support delivered via a face mask to provide either continuous positive airway pressure (CPAP) or intermittent positive pressure ventilation (iPPV), which will be collectively referred to as standard respiratory support (SRS). However, if this approach fails to support neonatal respiratory function, invasive intubation, and mechanical ventilation is required.

SRS is often insufficient to support very preterm neonates at birth. Preterm neonates often have poor respiratory effort at birth and as such are unable to fully aerate the lung. Pre-clinical studies have shown that this leads to non-uniform lung liquid clearance and increases the risk of hyperinflation injury in aerated lung regions during subsequent breaths/inflations. Given the rise in rates of prematurity globally and the life-long morbidity of neonatal lung injury, novel neonatal ventilation approaches have been investigated. This has led to the exploration of non-conventional strategies of ventilation, such as a sustained inflation (SI) (3). The use of a SI as a ventilation strategy in apnoeic neonates at birth was informed by early observational studies examining factors leading to functional respiratory capacity (FRC) formation in asphyxiated neonates (4, 5).

Although there is no internationally accepted definition of a SI, it has previously been defined as a positive pressure inflation applied over a longer time period than normally used in SRS. A SI aims to improve the uniformity of lung aeration (6–12) and the newborn cardiorespiratory transition (13, 14). A SI has not been shown to reduce lung injury, with some pre-clinical studies suggesting it may injure the lungs at high pressures (15, 16).

Despite many studies investigating the use of a SI in both animal models and human neonates, there is considerable variation in the use of the SI and newborn outcomes with recent studies questioning the benefits and safety of SI use clinically (17, 18). This narrative review aimed to synthesize the current animal and clinical literature providing a SI to newborns in the delivery room to gain a greater understanding of factors surrounding the delivery of a SI and newborn cardiorespiratory outcomes.

## Synthesis of Current Knowledge

### Search Strategy

**Figure 1:**
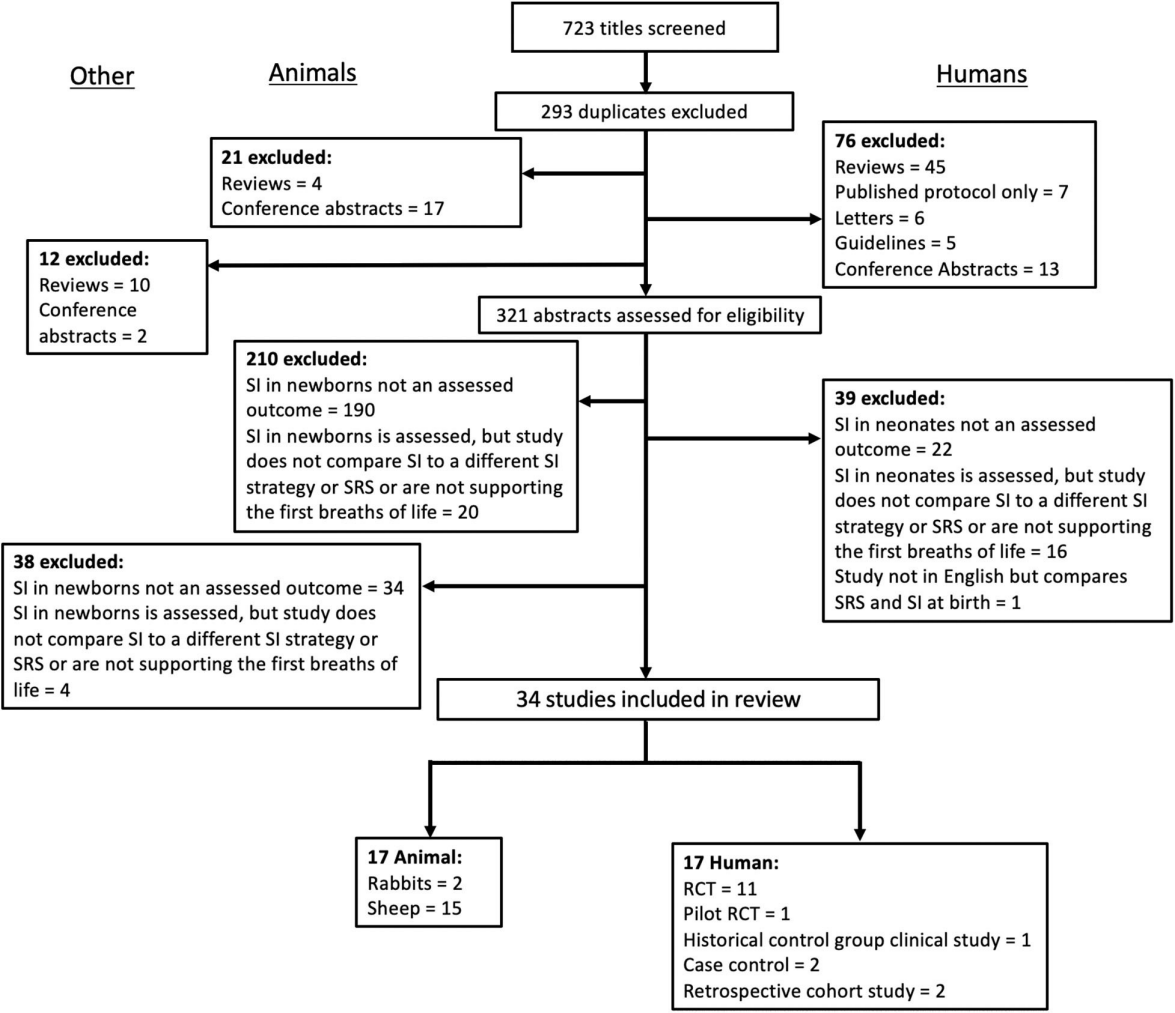
Summary of literature search and selection of studies included in the current synthesis of studies in animal and clinical literature evaluating the use of a sustained inflation on newborn cardiorespiratory outcome.

A systematic search of the literature was conducted to identify studies investigating the use of a SI in newborns in both animal and clinical studies. OVID Medline, Embase and Scopus were searched using the keywords “*sustained*,” “*inflation*,” “*infant, newborn, neonate*,” and the Map term to Subject Heading (MeSH) terms “*infant, newborn*,” “*infant, premature*,” “*fetus*,” “*sheep*,” “*rabbit*,” and “*animals, domestic*” (CJL). All studies published prior to April 2020 were included. Study titles and then abstracts were screened, with eligibility for inclusion based on full text assessment. Studies were included if they were original research studies published in English and compared the use of SI in newborns to either a SRS (CPAP or iPPV) or at least one other intervention to provide respiratory support for the first breaths of life (i.e., stepwise PEEP). The reference lists of included studies and review articles were examined to identify relevant articles not captured by electronic search. The combined search strategies yielded 723 studies, of which 34 were eligible for inclusion in this review (see Figure 1 for exclusion). Of these, 17 were animal experimental studies and 17 were clinical trials. The review was undertaken to highlight the inherent variability between and to tease apart methodological approaches and findings between the studies. This was conducted in the form of a narrative review following the systematic literature search as it was not appropriate to perform a meta-analysis due to heterogeneity between studies (19). For clarity, when discussing studies in this review all humans are referred to as neonates and animals are referred to by the species. Where sentiments from both animals and humans are discussed in combination, they are referred to generally as newborns.

### Tabulated Data

For each animal study, the publication characteristics (author, year, and species), SI parameters (length, pressure, number of SIs given, method of delivery) and experiment details (aim, physiological parameters measured, and outcome) were tabulated (Table 1). To facilitate comparison between animal and human studies in terms of gestational age and phase of lung development, animal gestational age at delivery was converted to human gestational age (Table 1) (6, 23, 26). As this review evaluates the effect of a SI on functional as opposed to anatomical lung differences, where appropriate comparison between gestational age in relevant animal studies have been converted relative to functional properties of the human lung (Table 1) (6, 27, 28). As these studies investigated multiple components of newborn physiology, the measured parameters were broadly characterized into blood gases, non-invasive oxygenation, lung mechanics, markers of blood flow and pressure (pulmonary and carotid artery), lung injury, and the use and type of dynamic lung imaging for each study (Table 1).

**Table 1:**
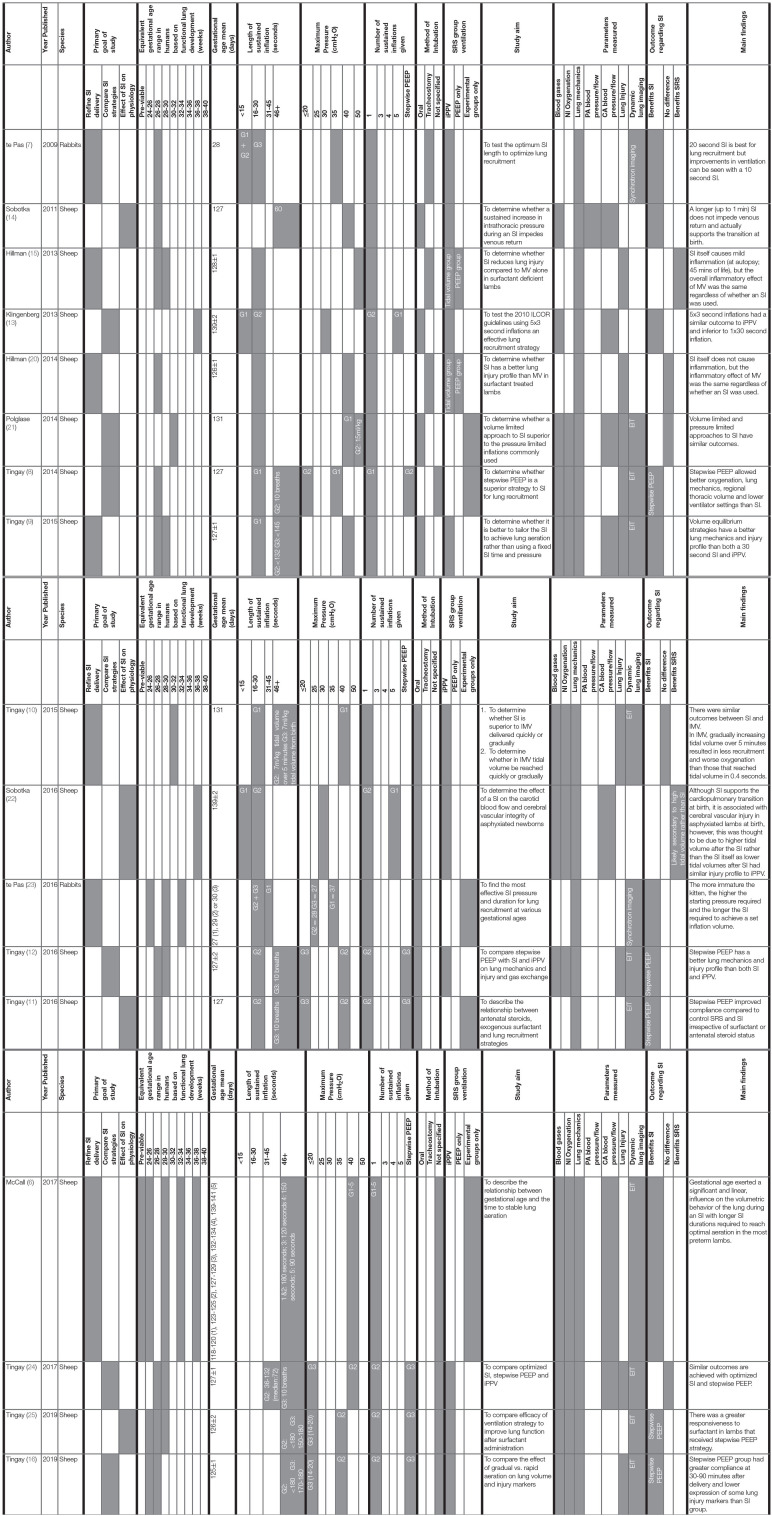
Animal studies investigating the effect of sustained inflation on newborn cardiorespiratory outcome.

*Individual squares are shaded to indicate gestational age of animal studies relative to comparative functional properties of the lung in the human neonate. Cumulative squares were shaded to indicate the length and pressure of sustained inflation where applicable. Where multiple groups were included within the study, the SI strategy used for each group is listed, prefaced with a G for group. Lung mechanics includes tidal volume, dynamic respiratory system compliance, airway pressures, and alveolar arterial difference in oxygen (AaDO_2_). Lung injury included any markers of lung injury, most commonly protein content in the broncho-alveolar lavage fluid and expression of messenger ribonucleic acid (mRNA) markers in lung tissue. Markers of cardiorespiratory transition included evaluation of both blood pressure and flow in the pulmonary artery (PA) and carotid artery (CA). Non-invasive (NI) oxygenation includes pre-ductal oxygen saturations (pre-ductal SpO_2_) and near-infrared spectroscopy (NIRS). Dynamic lung imaging includes electrical impedance tomography (EIT) and simultaneous synchrotron phase contrast X-ray imaging and plethysmography. Stepwise PEEP was listed in the outcomes regarding SI column when the benefit conferred from an SI strategy applied to stepwise PEEP only. Sheep, term = 147 days. Rabbit, term = 32 days. cmH_2_O = centimeters of water; IMV, intermittent mandatory ventilation; iPPV, intermittent positive pressure ventilation; MV, mechanical ventilation; PEEP, positive end expiratory pressure; SI, sustained inflation; SRS, standard respiratory support. Defined in all studies using stepwise PEEP as: begin at 6 cmH_2_O, increase after 10 breaths by 2 cmH_2_O until 20 cmH_2_O, then decrease by 2 cmH_2_O until 6 cmH_2_O except (8), where commencing PEEP was 4 cmH_2_O, increase after 10 breaths by 2 cmH_2_O until 20 cmH_2_O, then decrease by 2 cmH_2_O until 6 cmH_2_O. Summary of study demographics and variables in delivery of intervention to newborn animals and outcome in studies comparing sustained inflation to standard respiratory support strategy. Studies are presented in chronological order*.

**Figure 2:**
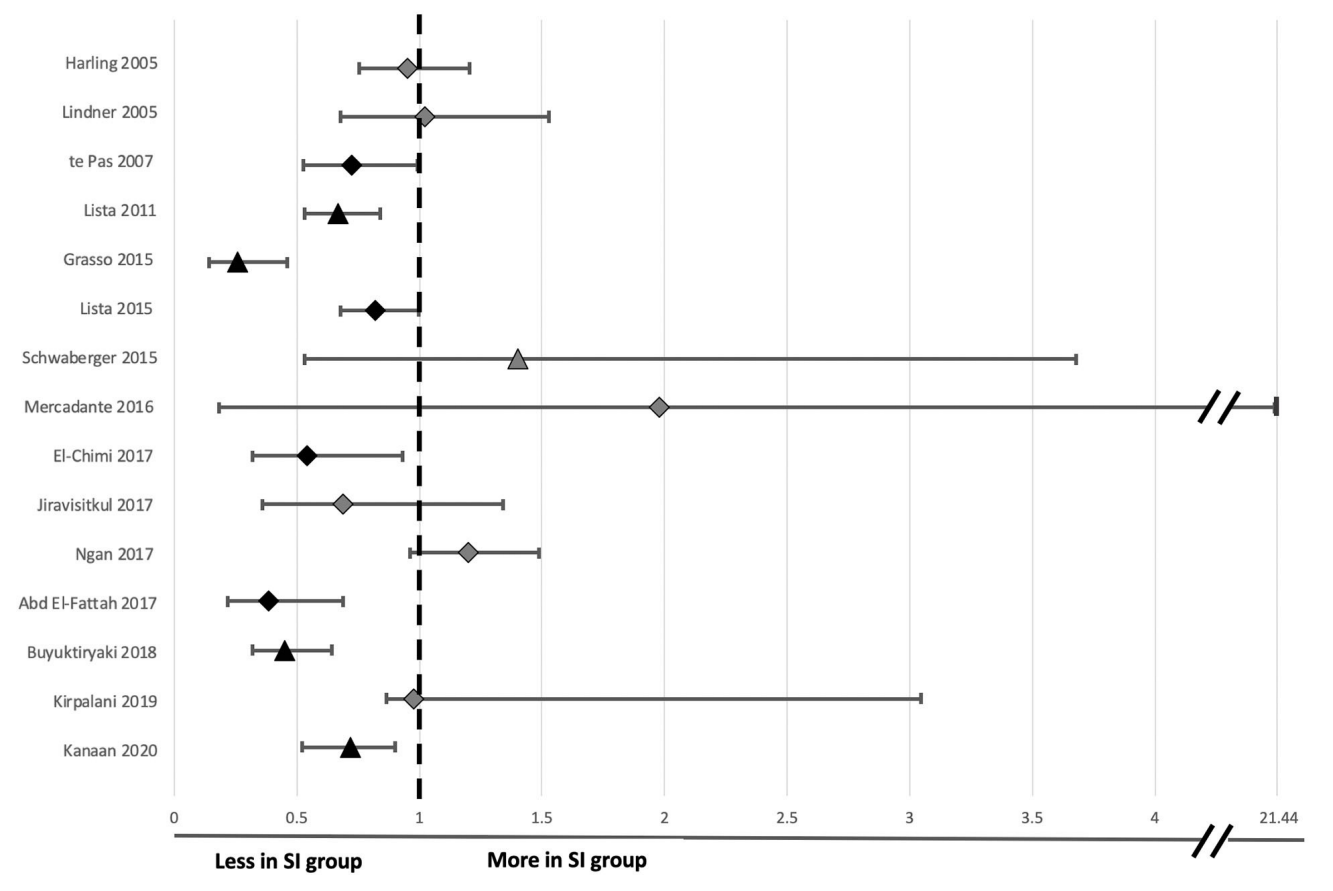
Forest plot of odds ratio and 95% confidence intervals for risk of endotracheal intubation following sustained inflation compared to standard respiratory support in clinical studies evaluated in Table 2. The timing taken into account for need for endotracheal intubation is noted for each individual study in Figure 3 except Kirpalani et al. at which is listed at 48 h following birth for consistency with other studies. Relative risk and confidence interval values were extracted from the 2020 Cochrane review where possible (45), or manually calculated (31–33, 40, 41, 44). Diamonds indicate RCTs and triangles indicate non-RCT study designs. Black indicates data sets resulting in significant differences and light gray indicates data sets reporting no significant difference in risk of intubation between the sustained inflation and relative control group within individual studies.

**Figure 3:**
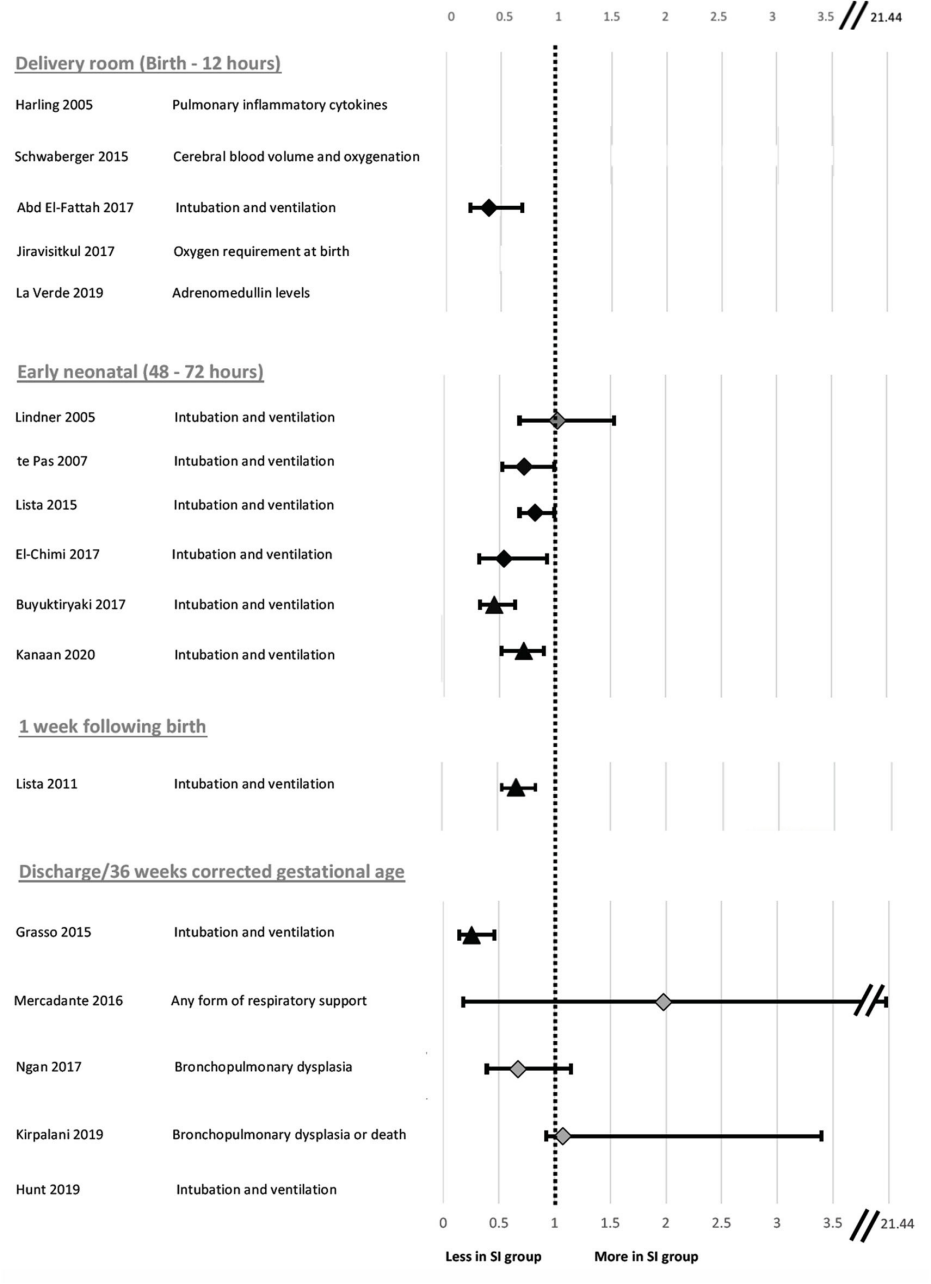
Forest plot of relative risk and 95% confidence intervals for primary outcomes reported in clinical studies evaluated in Table 2. The individual studies have been ranked based on timing of primary outcome evaluation. Relative risk and confidence interval values were extracted from the 2020 Cochrane review where possible (45) or manually calculated (18, 31–33, 40, 41, 44). Diamonds indicate RCTs and triangles indicate non-RCT study designs. Black indicates data sets resulting in significant differences and light gray indicates data sets reporting no significant difference in risk of primary outcome in SI compared to SRS. Where the primary outcome was a continuous variable, relative risk was unable to be calculated and a value has not been reported.

For each clinical study, the study characteristics (author, year and country of origin, study type), population characteristics (sample size, gestational age mean, and range), SI and SRS parameters (length, pressure, number of SIs given, type of SRS given, method of delivery, indication for delivery) and study outcomes (primary outcome, timing of primary outcome measurement relative to birth and primary outcome result) were tabulated (Table 2). The study measures and individual outcomes have been tabulated from data reported in the original publications. Reporting of overall study outcomes in text are based on the authors' interpretations of the overall original study outcomes. Where applicable, Cochrane review data was extracted to generate a relative risk (RR) and a 95% confidence interval (CI) for each included study (45). Where studies were not included in the Cochrane review, a RR and CI was calculated from raw data included in the original study authors' publication. These data were reported alongside authors findings (Table 2) and used to create a Forest plot for risk of intubation and mechanical ventilation to compare between studies (Figure 2) and also to rank primary outcome timing evaluated relative to birth (Figure 3). This was not possible for studies using continuous variables to measure primary outcome.

**Table 2:**
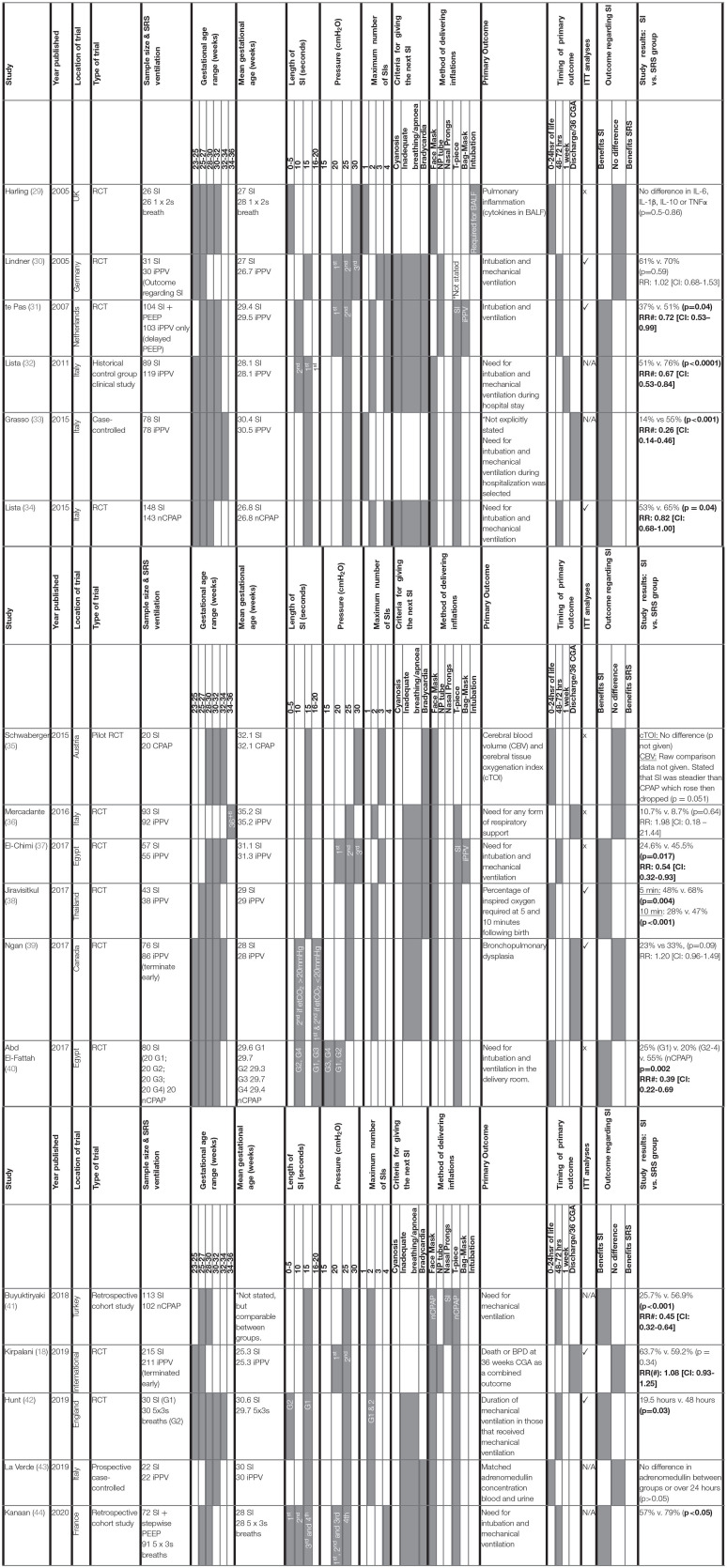
Clinical studies investigating the effect of a sustained inflation on neonatal respiratory outcome.

Squares are cumulatively shaded to indicate gestational age. Where squares are not shaded, information regarding this parameter was not described in the study. Where multiple SIs are given at different lengths or pressures, these are indicated in white text.

*BALF, bronchoalveolar lavage fluid; CGA, corrected gestational age; CI, confidence interval; cmH_2_O, centimeters of water; IL, interleukin; ITT, intention-to-treat; NP, nasopharyngeal; RCT, Randomized control trial; RR, relative risk calculated from Cochrane review (45). RR#, relative risk calculated by authors from incidence of intubation and mechanical ventilation in SI and SRS groups reported in individual studies; RR(#), relative risk calculated by authors from incidence of intubation and mechanical ventilation in SI and SRS groups however met criteria for inclusion into a Cochrane review; SI, sustained inflation; TNFα, tumor necrosis factor alpha. Summary of study demographics and variables in delivery of intervention to neonates and outcome in studies comparing sustained inflation to their respective standard respiratory support group. Studies are presented in chronological order*.

### Investigating the Effect of Sustained Inflation in Clinical and Experimental Literature

#### Findings From Animal Studies

Relevant study demographics are reported in Table 1 for the 17 included animal studies. These studies were published between 2009 and 2019. The large size of sheep makes them an appropriate animal model for complex physiological studies, with 15 studies using sheep. However, the function of sheep lungs is not consistent with anatomical maturity when compared to human lungs, and so gestational age conversion from sheep to human is based on similarity in functional maturity rather than anatomical maturity (Table 1) (6, 27, 28). Two studies used the smaller animal model of rabbits, which (1) have similar anatomical/functional properties at a given gestational age compared to humans and (2) are ideal for experiments using synchrotron imaging to assess lung aeration and respiratory mechanics at both the global and regional airway scale given their smaller size. Relative to the lambs, the rabbit studies have investigated outcomes after a relatively shorter duration following SI and to date have not assessed markers of lung injury. Conversely, only one observational study to date has investigated dynamic mechanics during SIs in a lamb model (46). Despite their differences, each animal model and investigative approach provides important knowledge to understand the effect of SIs on newborn physiology.

There is a fundamental difference in the weight of sample size and/or power calculation reporting considerations between clinical and animal studies. While the sample size of animal studies is powered to detect differences between groups in the main outcome, just as clinical studies are, animal studies can also minimize interacting variables and maximize treatment effects to identify differences between groups. Although this can limit the focus of the study to differences in the primary outcome in a highly controlled setting, it does avoid the use of inappropriately large animal numbers, which is one of the primary tenets of animal experimentation. As such, not all publications include a description of power calculations. In the animal studies included in this review, all studies reported sample sizes in each group. Specific power calculations were stated in methods or other justification in 8 studies (6, 9, 11, 12, 16, 23–25). One study included a partial statement (20) to justify smaller sample sizes for some groups, but no justification of the overall group sizes. While 8 studies did not report power calculation or sample size justification (7, 8, 10, 13–15, 22, 47). However, as the animal studies reported significant differences in at least one of their main outcomes, it is evident that the studies were powered appropriately to detect differences under the defined experimental conditions.

The overall goal of the animal studies was to refine the delivery of SI (7 studies), compare the effects of SI with a different ventilation strategy (6 studies) or examine the effect of SI combined with other factors upon newborn physiology (4 studies). There was variability in the gestational age range evaluated between studies, however, all studies investigated outcomes in premature newborns with uncomplicated pregnancies with the exception of 2 studies investigating outcome in a birth asphyxia model.

Studies differed in the type of SI strategies used, with 10 using variations on pressure-based strategies (7, 8, 10–15, 20, 22) and 8 using a volume-based strategy (6, 9, 10, 16, 21, 23–25). Six studies also included a stepwise positive end expiratory pressure (PEEP) group in addition to a SI group which have been explored in this review as they fulfilled the inclusion criteria (8, 11, 12, 16, 24, 25). While both SI and stepwise PEEP approaches are pressure-based interventions aimed at improving lung aeration, they target different phases of the respiratory cycle.

All animal studies either compared multiple SI ventilation strategies (a longer or physiological SI to shorter or fixed SI = 2 studies, stepwise PEEP strategy to SI = 6 studies, SI parameters in various gestational age cohorts = 2 studies, other SI group comparisons = 3 studies), or to a SRS group (iPPV = 10 studies, iPPV and PEEP alone as separate control groups = 2 studies; Table 1). These protocols differed in the length, pressure and number of SIs given. Within the pressure-based protocols, two studies used multiple (3–5 repeats), short (3–5 s) inflations, 10 studies used a single fixed SI (5–60 s) and 7 studies used a single SI with length determined by degree of lung inflation (physiological SI; 21–306 s). With regards to pressure, the 2 studies that used multiple, short inflations both used pressures of 30–35 cmH_2_O. The fixed SI studies commonly used pressures of 35–50 cmH_2_O while volume-based studies used temporarily higher pressures to create the SI (21). The physiological-based SI studies used pressures between 27 and 40 cmH_2_O and most of the stepwise PEEP protocols began at 6 cmH_2_O, climbing up to a maximum of 20 cmH_2_O before descending to 6–8 cmH_2_O. Of the animal studies that included a SRS group, either a PEEP of 5 cmH_2_O (5 studies) (7, 10, 13, 14, 22) or 8 cmH_2_O (7 studies) (9, 12, 15, 16, 20, 24, 25) was used. In contrast with the clinical studies, all animal studies administered the SI to sedated newborn animals that were either intubated orally (9 studies) or via tracheostomy (3 studies) to control for effects of spontaneous breathing, as their primary focus was investigating the effect of SI on the newborn cardiorespiratory physiology. Five studies did not report their method of intubation, however based on other studies by the same authors, it could be assumed that these lambs were intubated orally.

The advantage of using animal models is to permit the use of invasive methods to comprehensively investigate the effects of SI on newborn physiology. The animal studies had broad aims primarily focused on investigating the cardiorespiratory transition that were measured at discrete time points from birth to between 7 min and 8 h post-delivery (Table 1). Blood gases (12 studies), non-invasive oxygenation (10 studies), lung mechanics (17 studies), pulmonary blood pressure and/or flow (1 study), carotid artery pressure and/or flow (7 studies), lung injury (9 studies) and dynamic lung imaging using either simultaneous synchrotron phase contrast X-ray imaging and plethysmography (2 studies) or electrical impedance tomography (10 studies) were used to assess the cardiorespiratory transition using either two (5 studies), three (1 study), four (3 studies), five (5 studies), or six (3 studies) outcomes in total. Eight studies included evaluation of derived indices of gas exchange such as AaDO_2_ to provide information regarding blood gases and lung mechanics.

The animal studies found an overall positive effect of a SI compared to iPPV and PEEP alone on short term physiological outcomes (Benefits SI, physiological SI or stepwise PEEP = 11 studies; No difference = 4 studies; Benefits SRS = 2 studies; Table 1). More specifically, 6 studies have shown that a single 20–30 s inflation improves markers of the cardiorespiratory transition compared to shorter inflations given either alone or repeated up to three times. Stepwise PEEP has been reported by 5 studies to improve lung mechanics compared to a single SI (8, 11, 12, 16, 25), improve lung compliance and aeration in response to surfactant and cause less lung injury (25), however there is lack of data as to whether the prolonged inflation time of up to 5 min impedes venous return to the heart. Pressure-based SI strategies of up to 45 s have a similar lung injury profile to iPPV (15, 20) and do not impede venous return (14).

#### Findings From Human Studies

Relevant study demographics are reported in Table 2 for the 17 human studies included in this systematic narrative review. Of the included studies, 11 were randomized control trials (RCTs), one was a historical control group clinical study, one was a pilot RCT, two were case-control studies, and two were retrospective cohort studies). Studies were published between 2005 and 2020. As discussed in the recent Cochrane review investigating outcomes of sustained vs. standard inflations during neonatal resuscitation, RCTs had varying levels of bias in their randomization, all were unblinded for initial data collection and only half the studies blinded the researchers who analyzed trial endpoints (45). All RCTs were designed as superiority studies. All but one RCT reported power calculations. Those that reported power calculations were adequately powered apart from three studies, all of which were underpowered due to early termination of the study. For 2 studies, early termination occurred due to slow enrolment of participants (30, 39). Data for the remaining study showed an association between SI and early death at <48 h (18). The study that did not report a power calculation (37) was likely underpowered based on the number of participants of studies with a similar primary outcome (31, 34). Compared to the SI treatment group, the relative SRS groups were defined as receiving either iPPV (8 studies), CPAP (6 studies), or another ventilation strategy where the initial breath was more than 2 s (3 studies). It is important to consider the variation within the SRS groups regarding PEEP, as although iPPV given via a T-piece resuscitator is able to provide PEEP, iPPV delivered via bag-mask ventilation does not. Therefore, there may be differences in efficiency of respiratory support in the SRS groups between studies to support the newborn's lung function.

Gestational ages ranged from 23^+0^-36^+6^ weeks with mean gestational ages ranging 25.3–35.2 weeks. One study had a difference in the mean gestational age between groups of more than 7 days (29). With respect to degree of prematurity, 13 studies evaluated outcomes in extremely premature neonates (<28 weeks gestation at birth.), 14 studies evaluated outcomes in very premature neonates (<32 weeks gestation at birth), and 5 studies evaluated outcomes in moderate-late preterm neonates (32–36 weeks gestation at birth.). While a number of studies examined outcomes in a range of different gestational ages, only 1 study published results stratified by gestational age (44).

All clinical studies used pressure-based approaches to administer the SI, however, there was much variation in the times, pressures and number of SIs delivered. All studies delivered SIs for at least one duration (5 s = 3 studies; 10 s = 5 studies; 15 s = 13 studies; 20 s = 2 studies). Four studies administered at least two SIs of different durations. One study directly compared different SI strategies to each other. No studies stratified results based on the number of SIs delivered.

The SI pressure applied ranged from 15 to 30 cmH_2_O (15 cmH_2_O = 1 study; 20 cmH_2_O = 6 studies; 25 cmH_2_O = 14 studies; 30 cmH_2_O = 4 studies). There were differences between studies in the number of SIs delivered, with 2 studies administering 1 SI, 10 studies administering 2 SIs, 3 studies administering three SIs and 1 study administering 4 SIs. One study did not state how many SIs were administered (40). Increases in pressure on subsequent SIs were used in studies administering multiple SIs. Criteria for administering multiple SIs was based on a combination of one (3 studies), two (4 studies), three criteria (4 studies), or not stated (3 studies). These included cyanosis (4 studies), bradycardia (10 studies) and irregular breathing/apnoea (11 studies).

All clinical studies administered SIs via either face mask (13 studies), nasopharyngeal airway (3 studies), nasal prongs (1 study) or an unspecified, non-invasive method (1 study) unless intubation was required on clinical grounds as per the study protocol. One study used either a nasopharyngeal airway or face mask depending on the trial center (18).

The primary outcome varied greatly between studies. Respiratory insufficiency as indicated by the need for intubation and mechanical ventilation was the main outcome of 10 studies and presence of bronchopulmonary dysplasia was examined by 2 studies. Duration of mechanical ventilation, pulmonary inflammation, adrenomedullin levels in blood, and urine as a biomarker of pulmonary and vascular change during transition, death, oxygen requirement in the delivery room or markers of cerebral perfusion and oxygenation were all primary outcomes of 1 study each. One study listed need for any form of respiratory support as the primary outcome, however, it is unclear what interventions were considered respiratory support. One study had dual primary outcomes, however, the remaining studies each had one primary outcome. There was considerable variability within and between groups regarding the timing of primary outcome evaluation (Figure 3), including early markers in the delivery room or within 24 h of life (5 studies), early neonatal period (48–72 h, 7 studies), 1 week following birth (1 study) or later, either prior to discharge or at 36 weeks corrected gestational age (3 studies). One study did not state when data regarding need for mechanical ventilation was collected (33). Seven studies used intention-to-treat (ITT) analyses, however the short time between the respiratory support intervention provided, and outcome measurement mean that, although not explicitly stated, it is likely a further 4 studies were analyzed using ITT principles.

Eight studies showed that SI caused a statistically significant decrease in intubation and mechanical ventilation rates (Table 2; Figure 2 presents RR and 95% CIs for risk of endotracheal intubation ranked by study year where provided in text or able to be calculated from available data) compared to their respective SRS groups with improvements from 9 to 41%, likely inversely proportional to the rigor of the study design. One study showed that those given SI required shorter durations of intubation and mechanical ventilation and another showed less need for supplemental oxygen at 5 and 10 min post birth.

The largest RCT to date showed that although there was no difference in death or bronchopulmonary dysplasia (BPD) at 36 weeks between the SI and SRS group as a primary outcome, there was a statistically significant increase in death in the first 48 h of life in neonates given an SI (18). This led to early termination of the trial, thereby limiting definitive conclusions about the use of SIs in extremely preterm infants.

There were no significant differences between the SRS and SI groups in terms of markers of lung injury (29), cerebral tissue oxygenation (35), need for any form of respiratory support in moderate-late preterm neonates (30, 36), development of BPD as indicated by need for supplemental oxygen at 36 weeks (39) or adrenomedullin concentration in blood and urine (43). However, the studies investigating cerebral tissue oxygenation and development of BPD were not adequately powered to detect these differences. When the studies are ranked by timing of need for mechanical ventilation at birth (Figure 3), the most significant effect of SI was within 48–72 h post-birth. Nevertheless, a lack of difference in outcome in some studies indicates that a SI does not offer improved outcomes compared to the SRS interventions.

## Discussion

This systematic narrative review explores newborn SI studies in both animals and humans. In this synthesis, we have built upon previous meta-analyses to compare and contrast the different factors that influence SI delivery and outcomes in order to gain a more comprehensive analysis of these factors (17, 45, 48). In selecting studies for this review, no assessment of methodological quality was applied to any of the preclinical or clinical studies, and evidence grade was not used as a filter in the design to facilitate the broadest possible interpretation of the literature. Our tables include detailed information extracted from included studies to highlight strengths, limitations and differences between study designs and outcomes. A more comprehensive discussion of limitations and biases in SI meta-analysis literature has recently been discussed (17, 45).

**Figure 4:**
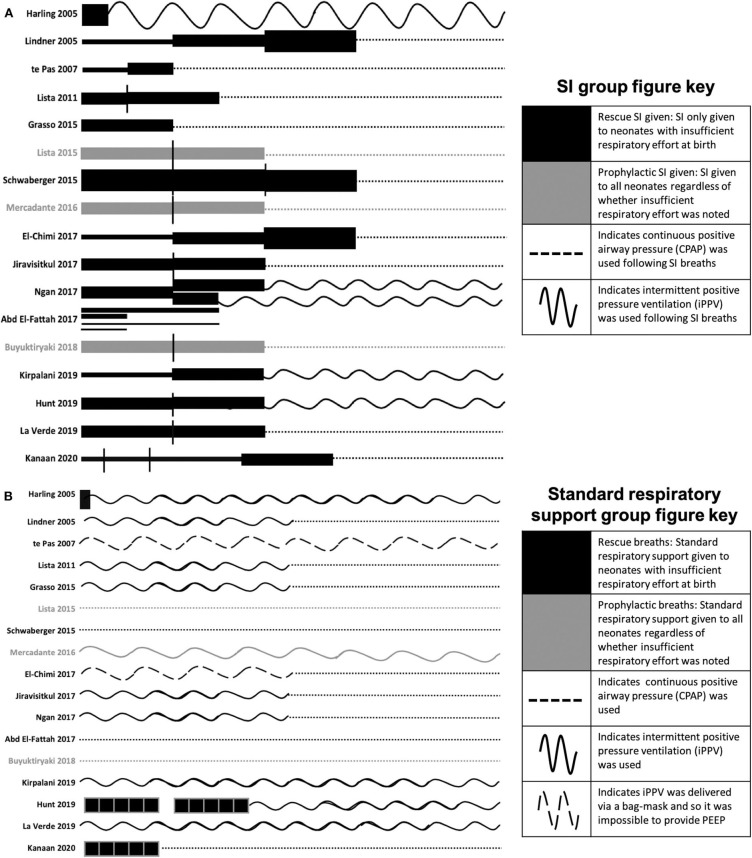
SI and subsequent respiratory ventilation protocols for the included studies **(A)** and SRS ventilation protocols **(B)**. The length of the line indicates the length of the SI and the thickness of the line indicates the pressure (1 cm = 0–5 s, 2 cm = 6–10 s, 4 cm = 11–15 s, 6 cm = 16–20 s; 1 pt = 15 cmH_2_O, 6 pt = 20 cmH_2_O, 14 pt = 25 cmH_2_O, 26 pt = 30 cmH_2_O). Where multiple SIs of the same pressure were given, vertical black lines separate each SI. Black lines indicate where the SI was only given to babies noted to have insufficient respiratory effort at birth. Gray lines indicate where SI was given to all babies regardless of whether insufficient respiratory effort was noted. Following SIs, dotted lines indicate continuous positive airway pressure was used, wavy lines indicate that intermittent positive pressure ventilation was used, dashed wavy lines indicate that intermittent positive pressure ventilation was delivered via bag-mask and so it was impossible to provide PEEP. To aid interpretation of the graph, neonates who did not respond to SI and subsequent continuous positive airway pressure or intermittent positive pressure ventilation have not been included as all studies further resuscitated and intubated neonates for intermittent mechanical ventilation as required. For Harling et al. the black line indicates a 2 s inflation. For te Pas et al. PEEP was not commenced in the control group until arrival in the neonatal intensive care unit (NICU) whilst PEEP was used throughout in the SI group. For Ngan et al. subsequent to the 1st SI, the length of the second SI was determined end tidal CO_2_. For El Fattah et al. four SI groups were included with various lengths and pressures of the SIs as per the graph. For Hunt and Kanaan et al. the black boxes indicate 5 × 2–3 second inflations. For Kanaan et al. ventilation protocol following SI and 5 × 3 s inflations are unclear, but it is noted that neonates were transferred to the NICU on either CPAP or NIV, so CPAP has been used in the graphs.

Animal models have allowed complex physiology surrounding the transition from fetal to newborn life to be investigated (Table 1). Clinical studies investigated primary outcomes related to death, respiratory, and cerebral function (Table 2). In clinical studies, we identified large differences in the way SIs and SRS were administered, which contribute to the heterogeneity observed in study outcomes. These include differences in pressure, time, number of SIs delivered, the type of SRS used and whether PEEP was used, and if respiratory support (SI or SRS) was only given to neonates who had insufficient respiratory effort at birth (rescue breaths), or if respiratory support was given to all neonates regardless of whether insufficient respiratory effort was observed (prophylactic breaths) (Figures 4A,B). A recent Cochrane review on the use of SIs found no significant differences between SI and SRS (45). The largest multi-center clinical trial to date found an increased rate of early death (<48 h post birth) in neonates who received SI, however there was no effect on rate of death at discharge or BPD (18). It is clear there is need for a greater understanding of the factors regulating the success of SI given the heterogeneity between the large clinical studies and the smaller clinical trials and animal studies. This is best gained from animal studies due to invasive testing required to determine these factors. Together these comparisons allow a greater understanding of the multiple factors influencing SI effectiveness and provide valuable insights to target future work in this area.

### Factors for Delivery of SI

#### SI Ventilation Strategy

Research into SI use in newborn resuscitation has investigated two strategies: (1) *pressure-based approaches*, where the SI is given based on a standard and pre-determined pressure with length either fixed or variable length depending on lung inflation; (2) *volume-based approaches*, where the SI is given on a predetermined tidal volume and length, with pressure adjusted to reach that tidal volume. Some studies combined approaches by using a tailored volume-based approach to determine the optimum pressure and length of a pressure-based approach (9, 23). By far, the most research exists for pressure-based strategies, with all human studies and 75% of the animal studies using variations on this strategy.

Pressure-based strategies with an adequate length (>10 s) and pressure (>15 cmH_2_O) are demonstrated in animal studies to improve aeration and support the cardiorespiratory transition (7, 14). However, the clinical studies suggest that SI use did not reduce the need for intubation and mechanical ventilation in two meta-analyses (17, 45).

Volume-based studies have only been used in pre-clinical animal studies to date (6, 9, 10, 16, 21, 24, 25). No specific guidelines exist regarding optimum volume delivery during SI, however early studies used a large volume initial breath (0–15 mL/kg) for a fixed pressure and duration (10, 47). Delivering a pure volume-based SI may have a similar outcome to a pressure-based SI, with a study in preterm lambs showing no difference in lung mechanics, arterial and cerebral oxygenation, blood pressure nor lung injury markers (21). While similar effects were observed when SIs were tailored by lung volume within the same study (21), it is important to recognize that the mechanical state of the preterm lung at birth should be considered, particularly with regard to liquid vs. air-filled lung and uniformity of aeration.

Further studies developed the use of a more physiological-based SI whereby a longer initial breath at a stable pressure and volume per breath was delivered, allowing the delivery of an individualized, larger volume of air due to the duration of the breath. This was devised following the understanding that every animal has unique ventilatory requirements and that the risk of over or underinflating the lungs present with a fixed volume strategy may be ameliorated with a physiological SI. All physiological-based SI studies to date use electrical impedance tomography (EIT) to achieve optimal lung mechanics. These studies have revealed large variations in the time taken to reach the optimum delivered volume between animals of a similar gestational age, however there is a trend to animals at younger gestational ages requiring longer duration breaths to reach the optimum lung volume (6, 10). A physiological-based SI may result in more uniform lung aeration and improved lung mechanics, however no differences have been observed between a physiological-based SI and pressure-based SI in terms of lung injury, oxygenation and markers of the cardiorespiratory transition (6, 9, 16, 25).

#### SI Length

In humans, 13 studies support the use of a 15 s SI. It is likely that this length has been chosen as 2015 ILCOR guidelines recommend 2–3 s inflations given five times simultaneously, leading to the assumption that a single 10–15 s SI may be equivalent and appropriate for clinical use (3, 13). A 10 s inflation is the minimum duration demonstrated to generate a statistically significant difference between those given SI and iPPV (31). However, the conclusion that a 10 s SI is statistically different to iPPV may have been confounded by the use of PEEP. In this study (31), SI was given via a T-piece, allowing PEEP to be administed and received early nCPAP in the delivery room, whereas the SRS group received iPPV alone via a bag-mask making it impossible to apply PEEP and did not receive nCPAP until arrival to the neonatal intensive care unit. Therefore, the benefit in the trial may have been due to all or one of the three components of the early support intervention group (Figures 4A,B).

In animal models, longer inflations of 20 s inflations are most commonly used. Use of this length was based on a landmark paper early in SI research that showed that a 20 s inflation improved lung mechanics more than a 5 or 10 s SI (7). However, 20 s was the longest SI investigated in that study, raising the possibility that a longer SI could be even more effective (7). Recently, the same group showed that the SI length and pressure required to reach target tidal volume at different gestational ages is higher than 20 s and trends toward an increase with earlier gestational ages in rabbits (23). These findings were confirmed by a similar study in sheep, however a longer median SI duration was used (6). The use of a 30 s SI has been investigated in 2 sheep studies and may be more beneficial compared to short SIs (≤5 s), but this has not been directly compared to a 20 s SI (9, 11–13, 22). The maximum length of a SI used to ventilate any animal in any study is 5 min, with a lower median SI length, which when given as an individually tailored regime, may ventilate the lungs more evenly and cause less lung injury than a shorter SI (6, 9). The study administering the longest SI duration investigated the effect of gestational age on time to achieve lung aeration and not clinical outcome (6). Studies aiming to describe clinically translatable outcomes typically used a SI duration of <3 min. It is likely that the ideal SI time in humans is at least 20 s, with neonates at younger gestational ages likely to benefit from a longer SI secondary to poor lung compliance due to incomplete lung liquid clearance in an immature lung (6, 23).

#### SI Pressure

The 2015 ILCOR guidelines recommend an initial inflation a pressure of 20–25 cmH_2_O for premature neonates and 30 cmH_2_O for term neonates for iPPV (3). Importantly the pressure needed to aerate the fluid-filled lung in neonates requiring resuscitation is greater than the air-filled lung, hence, after the initial breaths lower inflation pressures may be needed as the lungs aerate and compliance increases. This is reflected in the studies as all, but one clinical study used pressures of 20–30 cmH_2_O (Table 2). Animal studies on average used higher pressures, but it is unclear whether the differences are due to differences in respiratory mechanics between species. While the original SI studies used a SI of 35 cmH_2_O (7, 14), subsequent studies have up to 50 cmH_2_O, which may reflect different experimental conditions and equipment (Table 1). There is no evidence-based reason for differences between the pressures used in animal and human studies reported. In sheep, inflating pressures should not exceed 40 cmH_2_O, as using 50 cmH_2_O causes mild inflammation that is not present when 40 cmH_2_O is used (15, 20). One study directly compared the optimum pressure used to inflate lungs at various gestational ages in a rabbit model and suggested that the optimum pressure for extremely premature lungs was 37 cmH_2_O, very premature lungs was 28 cmH_2_O and moderately premature lungs was 27 cmH_2_O (23). This is consistent with the knowledge that earlier gestational age is proportional to decreasing lung compliance secondary to poor lung liquid clearance in apnoeic neonates. In addition, the pressure reaching the lung during non-invasive respiratory support may be impaired by upper airway (glottis) patency, preventing the pressure delivered reaching the lungs, particularly in newborns who are apnoeic (49). It also suggests that the lower inflating pressures recommended for premature neonates by ILCOR guidelines are unlikely to be high enough to achieve uniform lung aeration, however this needs to be balanced against the potential for increased lung injury (16, 50). From these studies, it can be determined that (1) higher inflating pressures than what are currently recommended are required for premature neonates depending on gestational age and (2) that these pressures should not exceed 40 cmH_2_O. Despite the observations from animal studies, these pressures must be validated in clinical trials before they can be applied to clinical management guidelines to prevent harm by overdistention, non-uniform aeration or lung injury.

#### Administering Multiple/Repeated SIs

Fourteen human studies repeated SIs, whereas in animal studies repetition was only performed if the SI was ≤5 s, giving a similar total duration of SI to that used in single SI studies (Table 2). Four of these studies were performed to test recommendations given by the 2010 European guidelines that a 3 s SI should be delivered 5 times (13, 22, 42). Two animal studies found that this short, repetitive ventilation strategy was similar to iPPV and may be worse than a single 30 s SI in physiological measures of the neonatal transition (13, 22). Two clinical studies found that a short, repetitive ventilation strategy resulted in statistically significant higher rates of neonates requiring intubation and longer duration of mechanical ventilation than SI (42, 44). All experiments fail to compare outcomes between the number of repetitions of SIs at an adequate length and pressure. Sub-group analyses were not performed in any of the human studies based on the number of SIs given, therefore it is difficult to infer the optimum number of times SI should be repeated.

#### Stepwise PEEP Ventilation Strategy

Stepwise PEEP is a ventilation strategy that aims to improve alveolar recruitment and aeration with normal tidal inflations during ascending and descending PEEP levels. Stepwise PEEP studies suggest improved lung mechanics, less lung injury and better response to surfactant compared to a fixed pressure-based SI strategy (8, 11, 12, 16, 25) and a similar or better effect than a physiological-based SI (16, 24). One study has been performed in humans to date and combined an SI and stepwise PEEP strategy with a maximum PEEP of 15 cmH_2_O (44). In the context of this review it is important to consider the physiological difference in ventilation approaches when comparing SI and stepwise PEEP maneuvers. The differences include (i) large differences in the duration (minutes) of the maneuvers, when the lung is exposed to elevated airway pressures, (ii) while inflation times are not prolonged during stepwise PEEP and are consistent with that used during SRS, mean airway pressures are elevated, (iii) while these approaches target different components of the respiratory cycle, the SI assumes that the expiratory phase is unnecessary because the lungs are liquid-filled and gas exchange cannot occur, and (iv) the inflating pressure during stepwise PEEP is the same or lower than SRS groups. It is clear that the relationship between pressure and time in these interventions requires greater interrogation toward optimizing the most physiological beneficial approach for the newborn.

There is lack of data as to whether the prolonged inflation time of up to 5 min impedes pulmonary blood flow and venous return to the heart. Some may argue that oscillating pressures between PIP and PEEP may reduce the impact of an SI on pulmonary blood flow and venous return. However, it has been observed in lambs that, during positive pressure ventilation pulmonary blood flow is reduced once PEEP levels increase to 8 cmH_2_O or higher (51). Pressure-based SI strategies showed a similar lung injury profile to SRS (15, 20) and did not impede venous return (14). Many lamb studies have investigated the effects of a pressure-based SI on cardiovascular parameters and found no significant effects, with 1 study (14) showing that a pressure-based SI facilitated the increase in pulmonary blood flow. While it is important to recognize that the effects of different SI interventions on pulmonary blood flow was not assessed in the rabbit studies, it is difficult to envisage why they would respond differently to lambs. Those studies focused exclusively on lung function but used similar pressures at either a similar or shorter duration than the lamb studies. When evaluating the effects of higher pressures and changes in PEEP on pulmonary blood flow it is important to consider the physiological difference between initiation directly at birth in a liquid filled lung vs. investigating pressure recruitment in an already aerated or partially aerated lung. The cardiovascular consequences of pressure-based maneuvers on the newborn lung is currently less well understood. However, it is important to consider the integral relationship between pressure and time targeted in respiratory support strategies to ensure that they maximize the benefits on improving lung aeration and oxygenation compared to risk of injury.

### Effect of SI on Newborn Physiology

#### Requirement for Mechanical Ventilation

Ten human studies used the primary outcome of requirement for intubation and mechanical ventilation (Table 2). Six of these studies showed that neonates who received SI were significantly less likely to require intubation and mechanical ventilation and one showed significantly shorter durations of mechanical ventilation in intubated neonates (31–34, 37, 40, 42, 44). The time of assessment of intubation and mechanical ventilation as an outcome varied between studies, ranging from the delivery room to 1 week after birth (Figure 3). However, in the largest study to date examining the most premature cohort of neonates, no differences were found in need for intubation and mechanical ventilation (18). A possible reason for these differences is that one quarter of the studies showing a significant decrease in intubation and mechanical ventilation were in, on average, older premature neonates given prophylactic rather than rescue breaths (Figure 4A). It is likely that for those neonates given prophylactic SIs, a portion of them may have been spontaneously breathing and therefore, the success of providing SI non-invasively by facemask was less likely to be impaired by a closed larynx preventing the inflation pressure reaching the lungs (52, 53). However, the trial looking at the oldest cohort of premature neonates showed no superiority of a SI compared to SRS, suggesting there is a “goldilocks range” of neonates in whom SI may have a beneficial effect because (1) they have sufficient respiratory drive to breathe spontaneously thus opening their larynx and allowing the SI to ventilate the lung and (2) their lungs are immature enough that they are unable to clear lung liquid and uniformly aerate the lung with the assistance of CPAP or iPPV alone. It is possible that this gestational age range is 28–34 weeks gestation at birth as statistically significant reductions in intubation and mechanical ventilation rates have been mostly described within this age range (31–34, 37, 40, 41, 44). Outside of this age range, no statistically significant differences in intubation and mechanical ventilation rates in studies providing rescue breaths have been described (18, 36), or in the 2 meta-analyses investigating all gestational groups as a whole (17, 45).

#### Cardiorespiratory Transition

Lung aeration triggers the initiation of the newborn cardiorespiratory transition by clearing lung liquid, which in turn reduces pulmonary vascular resistance and increases blood flow through the lungs (54). Two human studies have indirectly measured the cardiorespiratory transition (38, 43). The first, underpowered study of 81 neonates demonstrated that oxygen requirement was significantly lower in neonates receiving SI than CPAP at both 5 and 10 min after birth, indirectly indicating that SI supports the cardiorespiratory transition (38). The second study did not demonstrate a difference in adrenomedullin levels between groups at 24 h, and the authors suggested no difference between SI and iPPV on pulmonary and vascular changes during transition (43).

Fourteen of the included animal studies investigated the effect of SI on the cardiorespiratory transition at birth (6, 8–14, 21, 22, 24, 25, 55, 56). Stepwise PEEP, pressure-based and physiological-based SIs have been shown in multiple studies to improve lung mechanics compared to iPPV alone. In combination with PEEP, a SI creates FRC by facilitating more uniform lung aeration than iPPV or CPAP (7, 16). This is reflected in the higher pre-ductal oxygen saturations (SpO_2_) and lower partial pressure of carbon dioxide (PaCO_2_) in animals who received a SI, meaning that the lung is more efficient at exchanging gases when a SI is used (10). A SI has also been shown in animal studies to increase pulmonary blood flow following birth compared to iPPV, likely due to increased efficiency in lung aeration required to trigger the decrease in pulmonary vascular resistance, in turn increasing the amount of blood flow available for oxygenation by the lungs (14, 22).

#### Lung Injury

All strategies of ventilation have been shown to induce lung injury compared to uninflated lungs (16, 57). Three human studies used lung injury (via inflammatory markers or BPD) as a primary outcome and suggested that there were no statistically significant differences between SI and iPPV groups (18, 29, 39). A fourth, underpowered, prospective RCT investigated lung injury as a secondary outcome (40). This study suggested that neonates who received a SI for 20 s at 20 cmH_2_O had significantly higher lung injury markers immediately after intubation, however, these became similar to CPAP lung injury markers 12 h following intubation (40). It has been suggested that a repetitive SI strategy may be associated with increased risk of air leak development in the lungs, e.g., pneumothorax or pulmonary interstitial emphysema, however this is yet to be fully investigated (58).

Of the animal studies that analyzed lung injury, all but two suggested that the injury profiles between appropriate SI and iPPV are similar (8–10, 20, 21, 25). However, as the early response genes used to assess lung injury have an activation time course of only 60–90 min (57), it is possible that no difference was detected because lung tissue was collected up to 2 h after delivery. One study suggested that stepwise PEEP resulted in less lung injury than both SI and iPPV in the presence of surfactant and exogenous steroids in all groups (25). The second study was primarily focused on investigating lung injury and suggested that a SI caused more regional lung injury than either iPPV or stepwise PEEP in the youngest population of lambs (16). The SI in this study was delivered at 35 cmH_2_O until 10 s after the lung volume signal plateaued or for a maximum of 180 s. This study reported that even though the overall lung gas volumes (end-expiratory volumes and Vt) were similar, lung aeration was not uniform with greater aeration occurring in the non-dependent lung of the SI animals and dependent lung in stepwise PEEP animals. While this finding is not consistent with previous studies that have used imaging to measure the uniformity of lung aeration following a SI (1, 7), it is consistent with their finding of increased expression of injury markers following a SI in rabbits. However, in the context of lung injury, it is necessary to consider (i) the timing of the intervention provided, (ii) the pressure delivered and (iii) the timing of outcome evaluation relative to the intervention. Indeed, as the time course of activation of these early lung injury markers has a relatively short time window (15–90 min) (57), measuring tissue levels of these injury markers at 90 min after the intervention makes it difficult to assess whether the observed lung injury was directly related to the SI or to events that occurred after the SI. As the uniformity of lung aeration attributed to an SI can be lost during the first few inflations following the SI (7), the effect of a SI on post-SI ventilation needs further investigation. Indeed, it is necessary to understand differences in management both within and between study groups following the intervention in order to be able to attribute effects to the SI directly or ongoing post-intervention ventilation management itself as a potential source of lung injury (57). More recently, the advancement of proteomics approaches to assess lung injury have been investigated in preterm lambs receiving ventilation support for as little as 15 min up to 90 min (59), however these techniques have not been used in any studies investigating SI use to date.

Neither of the studies investigating birth asphyxia analyzed lung injury (13, 22), but have shown an increase in brain injury, thought to be due to greater and more rapid recovery from severe asphyxia. It is important to consider that while in immature lungs SI has a similar injury profile to iPPV, the effect of SI in other obstetric subpopulations with pathological causes of apnoea at birth is unknown. In future studies investigating SI use, there are a range of considerations that should be taken into account, specifically use of the appropriate power to detect changes in lung injury markers, adequate sampling regimens to reliably detect heterogeneous injury, the timing of lung injury marker collection and the relevance of the investigated pathways.

#### Brain Injury

Two studies have examined brain injury as an outcome of SI (22, 35). One pilot RCT assessed cerebral oxygenation and blood volume changes and found a statistically significant difference in blood volume patterns in the first 15 min after birth, but no difference in cerebral oxygenation (35). The authors suggested that this finding demonstrated that SI led to cerebral venous stasis due to impaired venous return to the heart. However, their findings that the SI group maintained a more consistent blood volume overall than the CPAP group may be attributable to cerebral autoregulation rather than cerebral venous stasis.

While SI does not cause cerebral venous stasis, in combination with a high tidal volume it has been observed to cause increased extravasation of plasma proteins at the blood-brain barrier in birth asphyxiated lambs compared to those given multiple, short inflations or iPPV (22). It must be noted that in this case of compromised, asphyxic lambs the authors attributed the increased extravasation to high tidal volume rather than the SI itself, as a SI in combination with a low tidal volume prevented any extravasation. It may therefore be concluded that while SI facilitates improved lung aeration and more consistent cerebral oxygenation, any respiratory interventions that followed must have a relatively low tidal volume (e.g., <7 mL/kg) to prevent brain injury through disruption of the blood-brain barrier. As this is the only study to investigate the effects of SI on asphyxic newborn lamb brains, further work is required to understand the mechanisms of action of SIs in compromised neonates.

#### Mortality

A recent RCT investigating the use of SI compared to iPPV on death or BPD among extremely preterm infants was ceased prematurely due to increased early mortality (within 48 h of life) in the neonates receiving SI as a prespecified safety variable (18). However, the absolute numbers of neonatal deaths were small (16 neonates in SI vs. 3 neonates in iPPV; 7.4 vs. 1.4%, respectively). Sixty-eight percentage of early deaths occurred in neonates aged 23–24 weeks gestation at birth and the statistically significant difference present at <48 h of life were no longer present at 36 weeks corrected gestational age.

There was no pattern regarding cause of death identified by the study authors and they noted that it could be a chance finding (18). It is important to consider that death was observed in the most vulnerable population of neonates (23–24 weeks gestation), with high baseline mortality rates [~35–65% (60)], due to the numerous potential complications these neonates face from extreme prematurity. This study highlights the importance of considering the impact timing of evaluation has on the overall primary outcome (Figure 3).

## Conclusion

This review has provided a comprehensive synthesis of factors influencing outcomes in both animal and clinical studies investigating the use of SI in newborns. However, we have highlighted many gaps in the literature, including the optimum SI pressure, time, method of delivery and number of repetitions as well as the effect of a SI on the brain, heart and other organs. The heterogeneity in newborn populations, SI administration and the timing of primary outcome makes direct comparison between clinical studies difficult (Table 2, Figures 3, 4).

Overall, some clinical studies have shown benefits of a SI relative to SRS to reduce the need for intubation and mechanical ventilation in neonates aged 28–34 weeks gestation at birth. It is possible that the optimum pressure of SI increases with increasing prematurity, with neonates born at earlier gestations requiring higher ventilation pressures. However, 2 recent systematic reviews and meta-analyses suggested that a SI should not currently be routinely used for preterm infants at birth (17, 45). The findings of the most comprehensive RCT in extremely premature neonates (<28 weeks gestation at birth) for early neonatal death within 48 h have raised concerns regarding the safety of SI use in the delivery room (18). The specific cause of death of these neonates and whether it was related to SI is unknown.

Given the inability to demonstrate long term benefit and as safety concerns have been raised, the use of SI in human neonates should be limited to clinical trials. However, this suggestion does not discriminate between intubated and non-intubated infants and therefore, assumes that these different subgroups of infants will respond similarly to a SI. Unfortunately, intubated infants have not been specifically examined, despite all preclinical evidence coming from intubated newborns and suggesting that they may benefit from a SI applied after intubation directly to the lung.

Taken together, the research indicates that while animal studies are required to inform the design of scientifically sound clinical studies, care should be taken to accurately translate the scientific findings. Animal studies have provided understanding of the complex physiology underpinning the effect of SI on the newborn cardiorespiratory transition. While the positive benefits of SIs in animal models have not been duplicated in clinical studies, the automatic assumption that this is an example of where humans respond differently to animals is problematic.

Instead, we should first ask why and what was done differently between the human and animal studies. In this instance, the answer is potentially straight forward, in that intubation is likely to be more efficient at transmitting the SI directly onto the lung as it bypasses the upper airway. Indeed, recent studies in animals have shown that the upper airway, particularly closure of the larynx, can block the airway and prevent air from entering the lungs (52). This highlights the need to encourage spontaneous breathing, so the larynx will open, to improve the success of non-invasively administered positive pressure ventilation, including a SI (52).

Another factor to consider is the efficacy of a SI in newborns with pathological causes of apnoea at birth and neonatal subpopulations with abnormal lung compliance and respiratory mechanics (e.g., congenital lung malformations, lung hypoplasia, infection, asphyxia). Although these populations were excluded from the clinical studies captured in this systematic search analysis, they may benefit greatly from SI, particularly if it is necessary to intubate them.

It is clear that future studies are required to optimize the factors related to SI use identified in this review and to close gaps in knowledge. Evaluation of the underlying physiology is required to identify the safest and most effective respiratory support to support the transition to neonatal life.

## Data Availability Statement

All datasets generated/analyzed for this study are included in the article/Supplementary Material.

## Author Contributions

CL, SH, AtP, and EM were responsible for the conception, design of the project, were involved in analysis, interpretation of the data, and drafted the article. CL and EM were each involved in data acquisition. All authors contributed to the final version.

## Conflict of Interest

The authors declare that the research was conducted in the absence of any commercial or financial relationships that could be construed as a potential conflict of interest.
